# Unmasking Turner Syndrome Via a Short Fourth Metacarpal in a Pediatric Wrist Injury

**DOI:** 10.1016/j.jpedcp.2026.200215

**Published:** 2026-05-08

**Authors:** Aoife O'Grady, Miriam Alcalá Ruiz, David Staunton, Paul O'Grady

**Affiliations:** 1 Department of Trauma and Orthopedic Surgery, Mayo University Hospital, University of Galway, Galway, Ireland; 2 Department of Pediatrics, Mayo University Hospital, University of Galway, Galway, Ireland

**Keywords:** Archibald's sign, incidental radiographic finding, pediatric wrist injury, short fourth metacarpal, turner syndrome

## Abstract

Turner syndrome is a chromosomal disorder associated with short stature, webbed neck, gonadal dysgenesis, and congenital cardiac anomalies. Subtle skeletal findings may provide diagnostic clues. We report a 12-year-old girl in whom an incidental short fourth metacarpal identified after wrist trauma led to the diagnosis of Turner syndrome.

Archibald's sign refers to the association between a short fourth metacarpal and gonadal dysgenesis.^1^ Since its original description, short metacarpals have been documented in various clinical conditions. Turner syndrome is one of the most common chromosomal disorders in individuals, with a typically female phenotype, and is characterized by complete or partial monosomy of the X chromosome, most commonly a 45,X karyotype. It affects approximately 1 in 2500 live female births.^2^

Clinical manifestations of Turner syndrome are heterogeneous but frequently include short stature, webbed neck, gonadal dysgenesis, and congenital cardiac anomalies. The more subtle skeletal features such as cubitus valgus deformity, nail dysplasia, or a shortened fourth metacarpal may be overlooked, especially when patients present for unrelated concerns.^2^^,^^3^

This case highlights how a routine evaluation for wrist trauma led to the radiographic detection of a short fourth metacarpal, ultimately resulting in the diagnosis of Turner syndrome.

## Case Report

**Figure 1:**
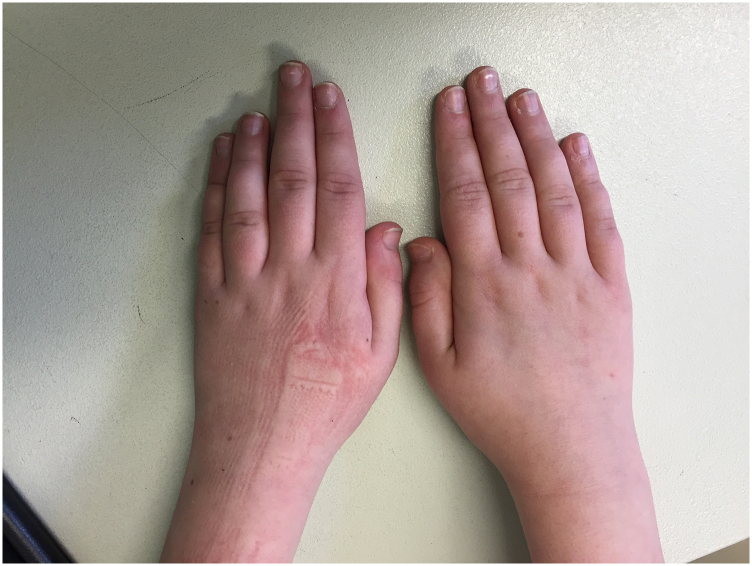
Clinical photograph of the hands showing shortening of the left ring finger.

**Figure 2:**
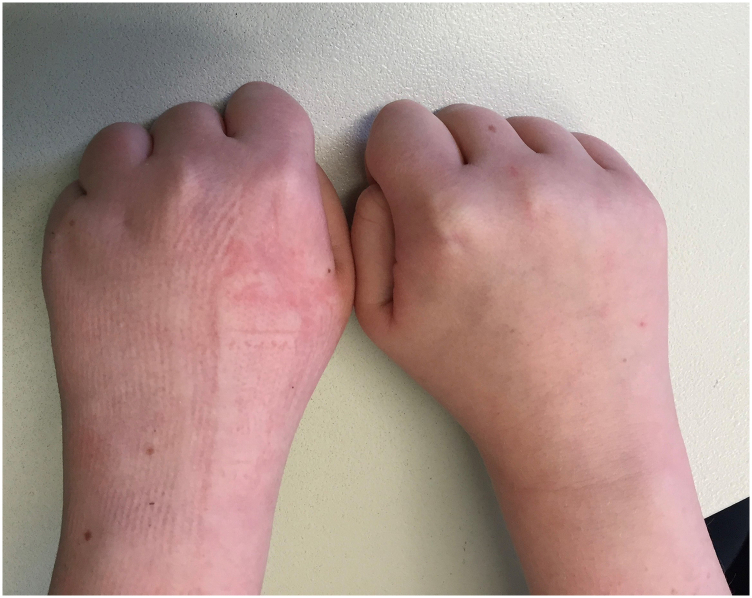
Clenched fist view. Archibald's sign.

**Figure 3:**
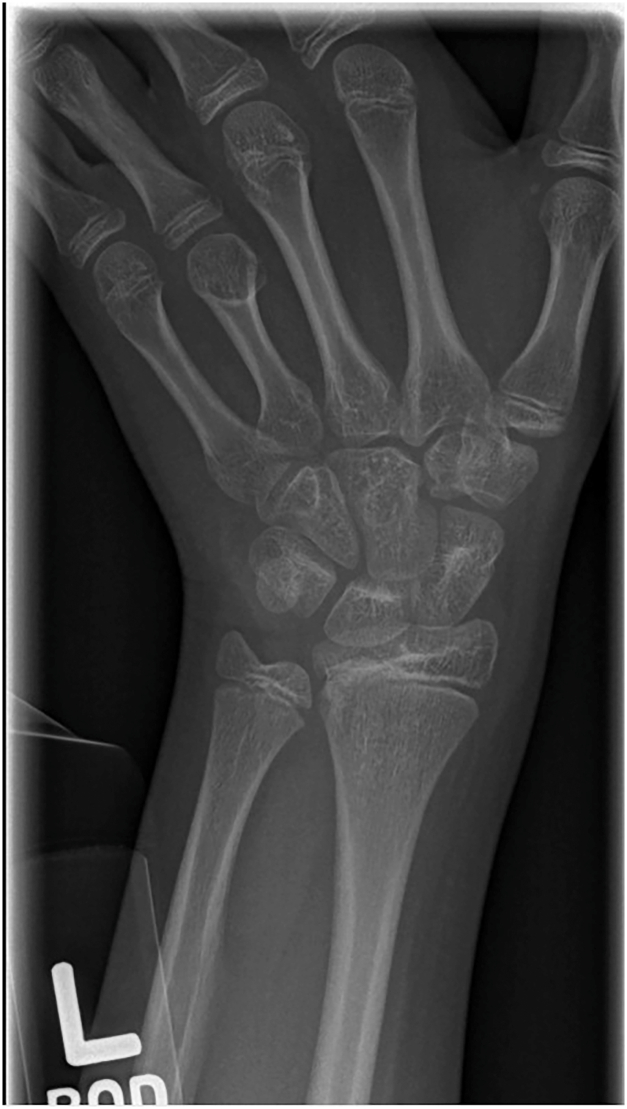
Radiograph of the left hand, demonstrating a short fourth metacarpal.

A previously healthy 12-year-old-girl presented to the pediatric emergency department after falling on her outstretched left hand during a school sports activity. Examination revealed tenderness and swelling over the distal left forearm. A short left ring finger was noted (Figure 1). With the hand clenched, the fourth metacarpal appeared shortened (Figure 2), and a pencil test confirmed a positive Archibald's sign. A radiograph confirmed a short fourth metacarpal (Figure 3). The right hand appeared normal. No neurovascular deficits were identified.

Plain radiographs confirmed a nondisplaced distal radius fracture and revealed a short fourth metacarpal. On further evaluation, a history of short stature was elicited, her height was at the 0.6th percentile (range, 0.4th-2nd percentile), and her weight was at the 9th percentile (range, 9th-25th percentile). Her mother described her as significantly shorter than her peers and family members. She had not yet entered puberty. There was no relevant family history.

Pubertal assessment showed Tanner stage I breast development and stage II pubic hair, with sparse, fair pubic hair and no axillary hair. Her skin was excessively dry, and a single hairy nevus measuring 4 cm × 1 cm was observed below her left knee. No other neurocutaneous stigmata were present.

Initial laboratory investigations showed normal urea and electrolytes, calcium, and parathyroid hormone levels. Vitamin D was within the normal range (58 nmol/L). Thyroid-stimulating hormone was elevated, whereas follicle-stimulating hormone and luteinizing hormone levels were markedly increased. Estradiol was <100 pmol/L, and progesterone was 0.7 nmol/L.

Karyotype analysis revealed a mosaic female karyotype: 1 cell line with monosomy X in 3 cells and another with 1 normal X chromosome and 1 pseudodicentric X chromosome. Electrocardiography demonstrated normal sinus rhythm, and cardiac echocardiography showed a structurally normal heart, with no bicuspid aortic valve and preserved biventricular function.

Based on the clinical and laboratory findings, a diagnosis of mosaic Turner syndrome (46,X with 1 pseudodicentric X chromosome/45,X) was made, associated with primary gonadal failure and short stature. The patient was started on growth hormone, thyroxine, and ethinylestradiol.

She was followed longitudinally. At her 2-year review, her growth velocity was 2.4 cm over 6 months. She had progressed to Tanner stage IV breast development, stage III pubic hair, and now had axillary hair. Pelvic ultrasound examination confirmed the presence of a pubertal uterus and bilateral ovaries.

## Discussion

The association between shortened metacarpals and endocrine disorders was first described by Archibald in 1959.^1^ The metacarpal sign is demonstrated radiographically by drawing a line tangential to the heads of the fourth and fifth metacarpals. The sign is positive if the line intersects the distal end of the third metacarpal rather than passing distal to its head. Clinically, this can be assessed using a pencil held across the flexed knuckles of the fourth and fifth fingers. Archibald observed that the sign appears more frequently in the left hand and carries greater diagnostic weight when seen in only 1 generation.^1^

Turner syndrome is often underdiagnosed due to its variable and subtle clinical presentation. Although classic features such as lymphedema, webbed neck, and congenital cardiac anomalies may be present, they are not universally observed.^4^ A shortened fourth metacarpal is a well documented but easily overlooked skeletal feature, resulting from premature epiphyseal closure.^5^^,^^6^ This finding may represent a normal anatomical variant or be associated with conditions such as prior trauma, pseudohypoparathyroidism, pseudopseudohypoparathyroidism, and multiple hereditary exostoses.^7^^,^^8^

Although the metacarpal sign lacks specificity, its identification in patients with additional suggestive features, such as short stature or delayed puberty, should prompt further investigation. In the present case, recognition of this finding on wrist radiography led directly to further clinical assessment and the diagnosis of Turner syndrome.

## Conclusions

“The hand is the visible part of the brain.” Although commonly attributed to Immanuel Kant, the origin of this quote is uncertain. In this case, the hand served as a visible clue to an underlying endocrine disorder. Identification of a short fourth metacarpal on wrist radiography prompted further assessment and led to the diagnosis of Turner syndrome, allowing appropriate endocrine management and progression of pubertal development. This case demonstrates that Archibald's sign can be a valuable clinical tool for the early recognition of Turner syndrome, particularly in borderline or atypical cases.

## CRediT authorship contribution statement

**Aoife O'Grady:** Writing – original draft, Project administration. **Miriam Alcalá Ruiz:** Writing – review & editing, Data curation. **David Staunton:** Investigation, Data curation. **Paul O'Grady:** Writing – review & editing, Conceptualization.

## Declaration of Competing Interest

The authors declare no conflicts of interest.
